# Psychological Outcomes and Predictors of Weight Loss in Adolescents With Severe Obesity Following a Reversible Endoscopic Bariatric Procedure

**DOI:** 10.3389/fped.2021.688287

**Published:** 2021-06-04

**Authors:** Simona Klemenčič, Ana Bujišić, Neža Štiglic Hribernik, Tadej Battelino, Matjaž Homan, Rok Orel, Primož Kotnik

**Affiliations:** ^1^Department of Endocrinology, Diabetes and Metabolism, University Children's Hospital, University Medical Center Ljubljana, Ljubljana, Slovenia; ^2^Community Health Centre Kranj, Kranj, Slovenia; ^3^Psychiatric Hospital Vojnik, Vojnik, Slovenia; ^4^Department of Pediatrics, Faculty of Medicine, University of Ljubljana, Ljubljana, Slovenia; ^5^Department of Gastroenterology, Hepatology and Nutrition, University Children's Hospital, University Medical Center Ljubljana, Ljubljana, Slovenia

**Keywords:** obesity, bariatric procedure, adolescents, psychological predictors, psychosocial functioning

## Abstract

**Introduction:** Adolescent and children obesity is a growing concern worldwide. Bariatric surgery is used as an effective treatment for adolescents with obesity and provides physical and mental health benefits. Application of alternative, minimally invasive, safe, and reversible endoscopic procedures, such as the Duodenojejunal bypass liner (DJBL), has been recently suggested as an effective treatment for adolescents with obesity. We explored specific psychological outcomes of adolescents with obesity during a year of follow-up after undergoing a reversible endoscopic bariatric procedure, and a year after removal. We were also interested in identifying psychological factors that could predict successful weight loss after the procedure.

**Methods:** Nineteen adolescent with severe obesity undergoing DJBL procedure were psychologically assessed in an open-label, prospective clinical trial (NTC0218393), at the implantation of device and at the removal of device after 12 months. Control group of 26 adolescents with severe obesity were recruited from the same outpatient clinic undergoing only conservative treatment. In addition, adolescents from the intervention group were followed for 12 months after the removal of the device. The Youth Self Report (YSR) was used to assess adolescents' emotional and behavioural problems; The Multidimensional Body-Self Relations Questionnaire (MBSRQ) to assess body image and The Eating Disorder Examination Questionnaire (EDE-Q) to assess attitudes and behaviours related to eating disorder.

**Results:** Significant improvements in somatic complain (*F* = 12.478, *p* = 0.001), emotional and behavioural problems (*F* = 7.169, *p* = 0.011) and food restraining (*F* = 9.605, *p* = 0.004) were found in the intervention group at device removal compared to the control group. Moreover, at the time of device removal compared to baseline, improvements in several psychological outcomes were found (*F* = 32.178 *p* = 0.000 for emotional and behavioural problems). Adolescents also became more satisfied with their appearance (*F* = 6.789, *p* = 0.019). Majority of observed changes remained stable at the next follow up a year after the device removal. Significant predictors of successful weight loss at device removal were fewer overeating episodes (*B* = 0.147, *p* = 0.022) and lower body satisfaction (*B* = 0.932, *p* = 0.013).

**Discussion:** Following a reversible bariatric procedure, improvements of psychological (emotional and behavioural) factors were found in adolescents with severe obesity. Psychological predictors of successful weight loss were identified, showing the greatest importance of eating behaviour and body satisfaction in successful weight loss.

## Introduction

Despite the fact that obesity prevalence in Slovenian adolescents stabilised recently (1), obesity remains a major worldwide health problem (2). In adolescents and children obesity is related to numerous health complications such as diabetes type 2, dyslipidemia, non-alcoholic fatty liver disease, kidney diseases, hormonal changes, and increased risk of premature death (3–9). Youth obesity has also been linked to multiple psychological comorbidities, such as poorer quality of life, emotional and behaviour problems (ADHD, ADD, etc.), social stigma, and self-esteem issues from a very young age and later in adolescence (3, 10–12). More depressive and anxiety symptoms (12, 13) and somatoform disorders were observed in children that were overweight compared to their peers (10, 14). Adolescents with obesity often not only have negative body image and poor self-esteem, but also lower academic and social self-image compared to their peers, which is even more evident in girls (11, 15). Self-esteem issues were related to weight-based teasing by peers and family members, which is frequently reported by children that are overweight or obese (16, 17). Furthermore, adolescents with obesity and lower self-esteem are more likely to engage in high risk behaviour such us smoking and consuming alcohol (11).

There is increasing evidence for bariatric surgery as an effective treatment for severe obesity in adults and also for adolescents (18, 19). Adolescents undergone gastric bypass showed similar weight loss, mortality rate, remission of type 2 diabetes, and hypertension as adults after 5-year follow up (19). However, most of the surgical bariatric procedures are irreversible and are related to acute and chronic complication (20). Concerning, the rate of abdominal reoperations was even higher among adolescents than among adults (19). Therefore, recent trends suggest application of less invasive, reversible endoscopic procedures, such as Duodenojejunal bypass liner (DJBL). DJBL is an endoscopically placed intestinal liner that can be removed at any time leaving original gastrointestinal anatomy intact. It limits the absorption of nutrients and leads to weight reduction and metabolic improvements (21, 22). DJBL treatment was found to be safe and effective, and therefore more appropriate for adolescents with severe obesity (23).

Previous research has focused mainly on physical and mental health benefits of more invasive bariatric procedures (24). From mental health perspective bariatric surgeries were found to be associated with sustained improvement in quality of life for adolescents with severe obesity, specifically in weight related quality of life and physical health related quality of life (25–27). Some research studies have also found that after surgical procedure adolescents showed significantly fewer symptoms of anxiety and depression and significantly improved self-concept compared to baseline (27, 28). Similar results were found among adults, in addition to improvement in body image, social functioning, and also improved eating behaviour (29–31). However, some studies showed that more than half of adolescents with psychopathology prior to surgery reported sustained elevated symptomatology 2 years after (32–34), moreover treatment for mental and behaviour disorders did not differ after 5 years of surgery (35). There is still a lack of evidence regarding mental health benefits of minimally invasive and reversible procedures such as reversible endoscopic procedures.

There is also a growing interest in identifying possible predictors of weight loss in obesity treatment. The existing studies have identified a variety of pre- and post-surgical predictors of weight loss, which remain inconclusive (36). Eating behaviour, specifically emotional and external eating have been found as negative predictors for weight loss 6 months after bariatric surgery in adults (37). Binge eating status has also been reported to predict poorer weight loss in some studies in adults (38, 39), however, others have reported that binge eating, assessed before surgery does not predict weight loss outcome (35, 40–43). In a recent study, neither anxiety nor depression levels significantly predicted successful weight loss, although patients achieving successful weight loss were characterised by lower mean scores of anxiety and depressive symptoms (44). Conversely, in other study adults with psychiatric disorders, including depressive disorder, had poorer outcomes in terms of weight loss 30 months after the bariatric surgery (43). Advancing age, high BMI, and cognitive impairments before surgery were also found as significant negative predictors for total weight loss in adults (37, 45).

Existing data about psychological benefits of non-surgically placed reversible devices for weight loss in adolescents are limited. Therefore, we aimed to evaluate the specific psychological outcomes of adolescents with obesity during a year of follow-up after undergoing minimally invasive reversible endoscopic bariatric procedure, and a year after removal. Especially we examined emotional and behavioural problems in adolescents, self-image and self-esteem, disordered eating attitudes, and behaviours based on the results of previous studies that adolescent with severe obesity have variety of psychological issues (10–14). We hypothesized that psychological burdens and negative eating patterns will decrease after bariatric procedure and remain relatively stable after bariatric procedure, as previously reported (28, 29, 37). The inclusion of control group of adolescents with obesity provided a critical comparison group.

Moreover, we aimed to identify the multiple psychological predictors of successful weight loss after the procedure. We hypothesised that adolescents with fewer emotional and behavioural problems, better self-image, and body satisfaction, would be more successful in weight loss due to more energy and coping strategies to follow instructions of bariatric team. Additionally, we predicted that adolescents fewer disturber eating attitudes and behaviours will achieve greater weight loss, according to results of other studies that examined effects of more invasive bariatric procedures (37–39, 46–48).

## Materials and Methods

### Participants and Procedures

Nine-teen adolescents with severe obesity who were undergoing reversible endoscopic bariatric procedure were followed in the prospective, single-arm, open-label study at University Medical Centre Ljubljana. The procedure with Duodenojejunal Bypass Liner Placement (EndoBarrier^®^, GI Dynamics, Lexington, MA, USA) was previously reported (49). The procedure was offered to all eligible patients between July 2014 and April 2017 that visited outpatient clinic and met the inclusion criteria: BMI > 99 percentile for age and gender or > 35 kg/m^2^, previously non-successful conservative treatment of obesity, age over 15 years, completed pubertal development, and at least one medical co-morbidity. Exclusion criteria were pregnancy (or pregnancy intention) and the following medical conditions: congenital gastrointestinal (GI) anomalies, previous GI surgery, inflammatory bowel disease, history of acute or chronic pancreatitis, coagulopathy, gallstones, severe GI reflux disease, active Helicobacter pylori infection, regular antithrombotic therapy, autoimmune connective tissue disorders. Prior to the procedure, all participants had complete psychological examination provided by the clinical psychologist to exclude patients with severe mental disorders (e.g., severe mood disorder, like major depression or psychotic disorders) or inability to follow instructions (e.g., intellectual disability).

Psychological evaluation was performed by a clinical psychologist for all participants with successful implantation at the time of implantation of the device T0 (*n* = 19) and at the removal of device 12 months after implantation (T1, *n* = 19).

Participants in the control group were recruited from the same outpatient clinic undergoing conservative treatment of obesity, after the intervention group was closed. Inclusion criteria were same as for the intervention group. Altogether 26 adolescents were followed up for up to 12 months. They received life style intervention by the same multidisciplinary team and were directed to the clinical psychologist to complete questionnaires (T0). Medical information for control group was obtained from medical records (T0). After 12 months (T1) participants from control group were asked to visit the clinic, where psychological examination and medical measures were taken again.

In addition, adolescents that underwent bariatric procedure were followed for 12 months after the removal of device—T2. Height, weight and general health status were also recorded for all participants at T0, T1, and T2 as previously described (49).

### Ethical Approval

The study was registered at www.clinicaltrials.gov (NCT02183935) and approved by The Republic of Slovenia National Medical Ethics Committee (#39/03/14) and by the Competent Authority. All participants and their parents signed informed assents/consent.

### Measures

#### Body Mass Index Standard Deviation Scores

Weight status was determined by Body Mass Index, calculated on a basis of measured height and weight data (kg/m^2^). Height and weight measurements were taken by a trained nurse using validated stadiometers and electronic digital scales. Both were rounded to the 10th decimal place. Body Mass Index Standard Deviation Scores (BMI SDS) were calculated according to reference curves (50).

#### The Youth Self Report—YSR (51)

The Youth Self Report—YSR (51) is a widely used youth self-report measure for the assessment of emotional and behavioural problems among youth ages 11–18 in the past 6 months. It is divided in two parts (1) competencies and (2) problems. It contains 119 items: 14 socially desirable items and 105 problem items. All of the items are short sentences worded in first person, to be answered on a 3-point scale: 0 = not true, 1 = somewhat or sometimes true, 2 = very true or often true. The YSR consists of the following eight scales: Withdrawn/Depressed, Anxious/Depressed, Somatic Complaints, Social Problems, Thought Problems, Attention Problems, Delinquent Behaviour, and Aggressive Behaviour. Withdrawn/Depressed, Somatic complaints and Anxious/Depressed together comprise a broad Internalising dimension, whereas Delinquent and Aggressive behaviours together constitute the Externalising dimension. Total Problems score can also be derived. Higher scores represent higher levels of problems. Good validity and test–retest reliability have been established (52).

#### The Multidimensional Body-Self Relations Questionnaire—MBSRQ-AS (53)

The Multidimensional Body-Self Relations Questionnaire—MBSRQ-AS (53) is a self-report inventory for evaluating attitudes related to body image. It is intended for use with adults and adolescents (15 years or older). It consists of 34 items that make up five subscales. Items are presented in a 5-point Likert format that range from 1 (very dissatisfied) to 5 (very satisfied). The subscales are Appearance Evaluation (7-item scale, high scorers indicating attractiveness/satisfaction with appearance, low scorers indicating unattractiveness/dissatisfaction with appearance), Appearance Orientation (12-item scale, higher scorers indicating more investment in one's appearance), Overweight Preoccupation (4-item scale, higher scorers indicating more anxiety and vigilance related to weight), Self-classified Weight (2-items reflecting subject's perception of own weight from very underweight to very overweight), and the Body Satisfaction scale (9-item scale assesses satisfaction/dissatisfaction with specific areas of the body). All subscales possess acceptable internal consistency and stability, internal consistencies were based on normative samples and were in range from 0.73 to 0.89 for females and from 0.70 to 0.88 for males (53). Test–retest reliability coefficients were obtained from samples of college students ages 18 years or over ranging from 0.74 to 0.91 for females and from 0.79 to 0.89 for males (53). Body Satisfaction scale has been recognised as acceptable assessment of body image (54), moreover clean factor structure and adequate convergent validity of MBSRQ-AS was confirmed in several studies (55–57).

#### The Eating Disorder Examination Questionnaire—EDE-Q (58)

The Eating Disorder Examination Questionnaire—EDE-Q (58) is a 28-item self-report questionnaire. It is used to assess disordered eating attitudes and behaviours over previous 28 days. This measure provides a Global score and four subscale scores: Restraint, Eating Concern, Shape Concern, and Weight Concern. Responses are on a 7-point scale, with higher scores reflecting greater eating-related pathology. Frequencies of disordered eating behaviours including binge eating, overeating episodes, and various compensatory behaviours are also assessed. The instrument has generally received support of its reliability and validity. Subscales has acceptable internal consistency (alphas ranging from 0.70 to 0.93) (59, 60) and test–retest reliability (ranging from 0.66 to 0.94) (60). There is inconclusiveness about EDE-Q factor structure (59, 61–63).

### Data Analysis

First, the analysis of covariance (ANCOVA) was used to determine the differences between two (treatment and control) groups (independent variable) for multiple independent variables, while still controlling for the starting value differences of the groups (64). The significance level was set for the two-tail hypothesis testing at α = 0.05. The analysis of variance (ANOVA) was used to determine significance of the changes over time in intervention group. Mann–Whitney test was used for assessing the between-group differences when comparing responding vs. non-responding participants.

Next, the multivariate linear regression was used, where the dependent value was BMI Change from 12 months (T1) and starting point (T0) and the independent values were psychological variables. Before the analysis, all independent variables were centred. The creation of the linear regression model was done in accordance with the backward stepwise procedure, where we started with the model with all the main effect terms and all the combinations between pair variables as the interaction terms. Then the procedure of the removal of the terms was conducted, where the terms of a higher order with the highest statistical significance (*p*-values) were removed from the model and the model was re-evaluated with the calculation of Akaike Information Criterion (AIC). This procedure of backward stepwise removal of the terms continued if the AIC metric was decreasing, and the resulting model was one with the lowest AIC as the stepwise procedure finished. The logistic regression with two-tailed hypothesis testing and the significance level α = 0.05 were used in the analysis.

## Results

Sample characteristics (age, gender and BMI SDS) of intervention and control group are presented in Table 1 at baseline (T0), after 12 months (T1), and 12 months after the device removal (T2). Descriptive statistics of self-reported psychological outcomes are presented in Table 2. In the intervention group all participants participated at T1, while in control group 16 participants responded (drop-out rate: 38%). There were no significant differences in age, BMI, and all measured psychological characteristics between participants who responded and the one who did not in control group. Twelve participants from intervention group responded at T2 (dropout rate: 29%). Participant who did not responded did not differ from the ones who did according to age, BMI, and most of the measured psychological variables, except for the higher scores in Thought Problems at T0 (*MR* = 14.50 vs. 7.38, Mann–Whitney *U* = 10.500, and *p* = 0.005) and higher scores in Restraining at T1 (*MR* = 13.36 vs. 6.73, Mann-Whitney *U* = 8.000, and *p* = 0.004).

**Table 1:** Sample characteristics for intervention and control group at baseline (T0), after 12 months (T1), and 12 months after the device removal (T2).

	**EB group**			**Control group**	
	**T0**	**T1**	**T2**	**T0**	**T1**
*n*	19	19	12	26	16
Age, ***M*** (*SD*)	**17.23** (1.24)			**16.09** (1.32)	
Gender (female)	12	12	6	13	11
BMI, ***M*** (*SD*)	**3.71** (0.31)	**3.21** (0.42)	**3.42** (0.37)	**3.35** (0.48)	**3.37** (0.64)

**Table 2:** Measure outcomes at baseline (T0), after 12 months (T1), and 12 months after the device removal (T2); and between group and within group differences for psychological variables.

		**EB group**		**Control group**		**ANCOVA (EB group vs. control group)**				**ANOVA (EB group)**			
	**T0 (*n* = 19)**	**T1 (*n* = 19)**	**T2 (*n* = 12)**	**T0 (*n* = 26)**	**T1 (*n* = 16)**	Between group differences (T0–T1)^*^		Baseline measurements (T0)^**^		**T0 vs. T1**		**T0 vs. T2**	
	***M* (*SD*)**	***M* (*SD*)**	***M* (*SD*)**	***M* (*SD*)**	***M* (*SD*)**	***F***	***p***	***F***	***p***	***F***	***p***	***F***	***p***
**YSR**													
Anxious/depressed	**7.37** (6.16)	**5.26** (6.40)	**3.90** (4.25)	**5.25** (4.83)	**4.50** (3.20)	1.834	0.185	56.744	**0.000**	8.247	**0.010**	10.557	**0.010**
Withdrawn/depressed	**3.74** (2.85)	**2.79** (2.59)	**2.90** (2.56)	**5.17** (3.80)	**4.13** (2.28)	0.567	0.457	22.868	**0.000**	4.726	**0.043**	0.112	0.745
Somatic complaints	**3.89** (4.04)	**1.58** (1.68)	**1.80** (2.62)	**2.46** (2.40)	**3.69** (3.26)	12.478	**0.001**	10.923	**0.002**	7.703	**0.012**	2.609	0.141
Social problems	**4.63** (2.94)	**2.81** (2.95)	**2.00** (1.87)	**3.17** (2.60)	**3.50** (2.58)	2.472	0.127	7.470	**0.011**	9.121	**0.009**	14.253	**0.007**
Thought problems	**3.42** (3.73)	**1.95** (1.75)	**2.30** (2.91)	**2.04** (1.97)	**2.50** (2.03)	1.743	0.196	3.074	**0.089**	3.111	0.095	1.000	0.343
Attention problems	**6.37** (5.16)	**5.63** (4.72)	**4.60** (4.27)	**5.71** (3.06)	**5.13** (2.31)	0.004	0.950	65.060	**0.000**	2.025	0.172	5.000	**0.052**
Rule—breaking behaviour	**4.47** (4.16)	**3.79** (2.88)	**3.90** (2.85)	**4.25** (2.47)	**4.13** (2.92)	1.057	0.312	41.859	**0.000**	1.454	0.243	3.857	0.081
Aggressive behaviour	**7.84** (3.70)	**6.84** (3.61)	**5.70** (4.03)	**6.58** (3.88)	**5.50** (2.76)	0.015	0.903	34.373	**0.000**	2.631	0.122	5.597	**0.042**
Internalising problems	**15.00** (10.98)	**11.68** (12.23)	**8.60** (7.29)	**12.88** (9.94)	**14,.88** (12.95)	2.265	0.142	14.926	**0.001**	4.362	**0.051**	5.862	0.039
Externalising problems	**12.68** (7.17)	**12.89** (9.04)	**9.80** (6.37)	**10.83** (5.63)	**12.25** (11.75)	0.075	0.787	4.123	**0.051**	0.011	0.916	13.071	**0.006**
Total problems	**49.68** (25.35)	**37.37** (22.15)	**32.30** (17.99)	**40.33** (19.50)	**38.69** (16.45)	7.196	**0.011**	80.304	**0.000**	32.178	**0.000**	9.551	**0.013**
**MBSRQ**													
Appearance evaluation	**2.24** (0.82)	**2.76** (0.70)	**2.72** (0.67)	**2.73** (0.79)	**2.71** (0.91)	0.595	0.447	7.956	**0.009**	6.789	**0.019**	3.291	0.113
Appearance orientation	**3.24** (0.60)	**3.34** (0.52)	**3.16** (0.53)	**3.24** (0.70)	**3.18** (0.62)	0.077	0.783	14.164	**0.001**	0.231	0.637	1.482	0.263
Overweight preoccupation	**2.96** (0.69)	**3.13** (0.77)	**3.16** (0.81)	**2.78** (0.89)	**2.53** (0.46)	3.741	0.063	2.679	0.113	0.025	0.875	0.079	0.787
Self-classified weight	**4.74** (0.36)	**4.58** (0.61)	**4.55** (0.44)	**4.40** (0.57)	**4.50** (0.55)	0.204	0.655	9.899	**0.004**	1.000	0.332	2.032	0.197
Body satisfaction	**2.70** (0.50)	**2.87** (0.67)	**2.74** (0.77)	**3.04** (0.58)	**3.06** (0.72)	0.031	0.862	9.238	**0.005**	1.914	0.185	1.116	0.326
**EDE-Q**													
Restraint	**1.59** (0.94)	**2.16** (1.09)	**2.20** (1.57)	**1.75** (1.02)	**1.12** (0.87)	9.605	**0.004**	0.012	0.912	3.028	0.101	0.483	0.510
Eating concern	**1.18** (1.20)	**0.77** (0.73)	**1.16** (0.99)	**1.01** (0.96)	**0.83** (1.05)	0.081	0.778	2.424	0.130	1.705	0.210	1.371	0.280
Weight concern	**3.22** (1.22)	**2.98** (1.04)	**3.10** (1.36)	**2.70** (1.31)	**2.35** (1.23)	3.053	0.091	36.769	**0.000**	0.746	0.400	1.465	0.265
Shape concern	**3.25** (1.45)	**2.91** (1.36)	**2.97** (1.45)	**2.76** (1.48)	**2.33** (1.44)	1.051	0.314	32.173	**0.000**	2.928	0.106	3.124	0.120
Overeating episodes	**3.59** (6.92)	**1.11** (1.68)	**1.22** (1.20)	**2.83** (4.68)	**2.14** (2.04)	1.497	0.235	0.001	0.975	1.967	0.180	0.401	0.550

*EB Group, intervention group with device implantation; YSR, The Youth Self Report; MBSRQ, The Multidimensional Body-Self Relations Questionnaire; EDE-Q, The Eating Disorder Examination Questionnaire; Statically significant p-values appear in bold text*.

* *Differences between experimental and control group after 12 months (T0-T1)*.

** *Differences between experimental and control at the baseline (T0)*.

### Improvements of Psychological Variables in Time

According to ANCOVA (Table 2), there were significant improvements in somatic complains (YSR), total emotional and behavioural problems (YSR), and food restraining (EDE-Q) in the intervention group at device removal compared to the control group (T0–T1). Those improvements were observed regardless of statistically significant differences between intervention and control group at baseline measurements.

In the intervention group, ANOVA showed statistically significant improvements in several psychological outcomes at the time of device removal compared to baseline (T0 vs. T1) namely Anxious/Depressed, Withdrawn/Depressed, Somatic Complaints, Social problems, Internalising Problems, Total emotional and behavioural problems (YSR). Moreover, adolescents became more satisfied with their appearance (MBSRQ). No improvements were found in variables referring to behaviours of eating disorders (EDE-Q). In addition, majority of observed changes remained stable at the next follow-up a year after the device removal (T0–T2).

**Figure 1:**
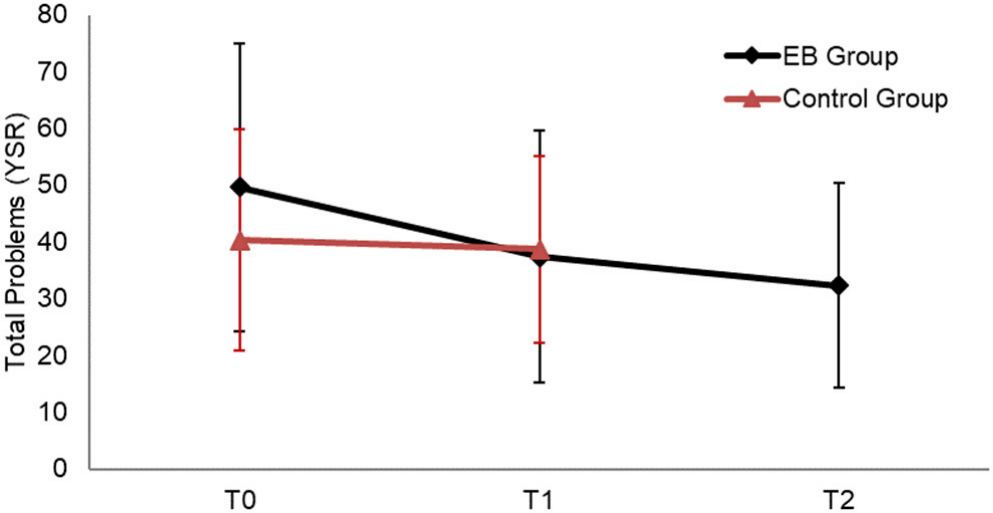
Total emotional and behavioural problems at baseline (T0), after 12 months (T1), and after 24 months (T2) for experimental and control group.

The main statically significant improvement in Total emotional and behavioural problems (T0–T2) for experimental group (compared to the control group) is presented in Figure 1. Figure 1 indicates that Total problems in the experimental group improved after the intervention (T1) compared to baseline (T0) of control group and remained stabled a year after removal.

### Psychological Predictor of Weight Loss

Table 3 contains the final multivariate linear regression model predicting weight loss. Weight loss (BMI SDS Change) was calculated by subtracting BMI SDS_T0_ from BMI SDS_T1_ (lower BMI SDS Change indicating greater weight loss). The final model achieved adequate fit (global predictive capacity equal to *r*^2^ = 0.725, adjusted *r*^2^ = 0.511, *p* = 0.046). The model indicated that the significant predictors of successful weight loss at device removal were fewer overeating episodes and lower body satisfaction.

**Table 3:** Multivariate linear regression model indicating predictors of weight loss (BMI SDS Change).

**Names**	***B***	**Exp *B***	***SE***	***t***	***p***
(Intercept)	−0.051		0.145	−0.354	0.732
**Body Satisfaction (MBSRQ)**	**0.932**	**1.458**	**0.301**	**3.097**	**0.013**
Withdrawn/Depressed (YSR)	0.052	0.440	0.033	1.562	0.153
Restraint (EDE-Q)	0.223	0.649	0.113	1.976	0.080
**Overeating Episodes (EDE-Q)**	**0.147**	**3.163**	**0.053**	**2.773**	**0.022**
**Withdrawn/Depressed** **×** **Overeating Episodes**	**0.044**	**0.458**	**0.014**	**3.131**	**0.012**
**Body Satisfaction** **×** **Overeating Episodes**	**0.292**	**0.374**	**0.117**	**2.497**	**0.034**
**Restraint** **×** **Overeating Episodes**	**0.086**	**0.252**	**0.036**	**2.397**	**0.040**

*B, regression coefficient; Exp B, standardised regression coefficient; SE, standard error of coefficient; t, t-value; p, level of statistical significance. Values of variables considered as statically significant appear in bold text*.

**Figure 2:**
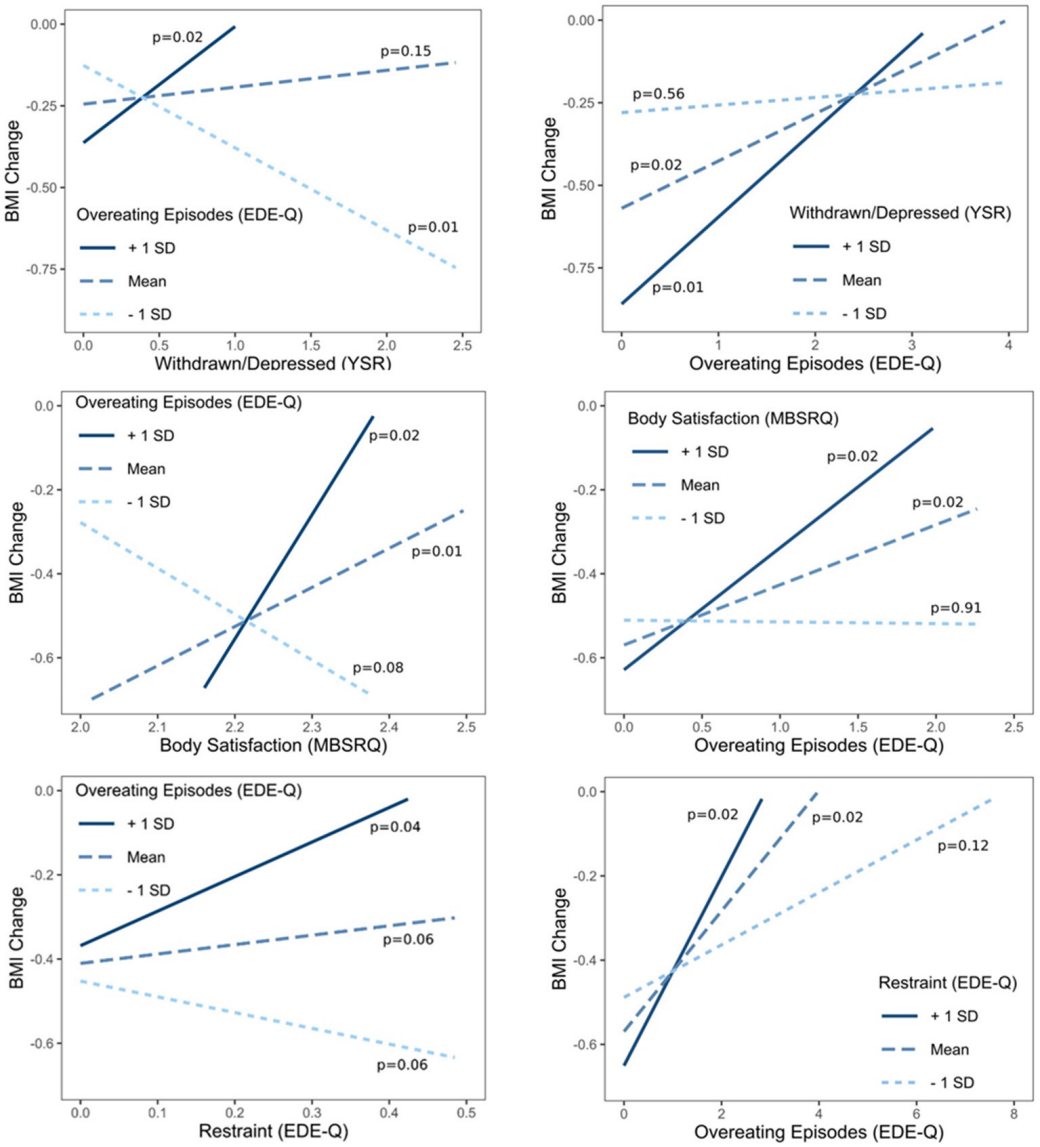
Simple slope analysis for three statistically significant interactions in the multivariate regression model.

Three interactions between independent variables demonstrated statistical significance, namely interaction between depression-withdrawn and overeating episodes (1), interaction between body satisfaction and overeating episodes (2), and interaction between restraining and overeating episodes (3). Simple slope analyses were calculated for those three interactions and are presented in Figure 2.

Simple slope analysis presented in Figure 2 shows that adolescents with the highest number of overeating episodes lost the least amount of weight, which was especially evident for those who reported more depressive symptoms (1), were more satisfied with their body (2) and reported higher levels of restraining with food (3). In other words, adolescents who reported more depressive symptoms (1), were more satisfied with their body (2) and reported higher levels of food-related restraining (3), lost more weight if they reported fewer overeating episodes.

## Discussion

In this preliminary and exploratory study, we aimed to investigated the specific psychological outcomes in adolescents with severe obesity undergoing reversible endoscopic bariatric procedure. We observed, according to our expectations, significant improvements in somatic complaints, total emotional and behavioural problems, and food restraint after 12 months of treatment compared to control group. Furthermore, within the experimental group additional improvements of depressive and anxiety symptoms and social problems were found. Adolescents were also more satisfied with their appearance. A year after device removal adolescents reported fewer externalising problems (especially aggressive behaviour) compared to baseline. Our results are comparable with studies investigating psychological outcomes of more invasive bariatric procedures that observed reduction in self-reported symptoms of anxiety, depression, anger and disruptive behaviour, and improved self-concept (27, 28). This can be explained with the growing feeling of competence after weight loss (65). Changes in psychological variables are considered favourable, are comparable with more invasive procedures, and are relatively stable a year after the procedure was finished, showing long term psychological effects of the treatment regardless of weight gain observed in some adolescents. Contrary to our hypothesis, we did not find changes in disordered eating attitudes and behaviours which could potentially contribute to weight gain. This is in contrast to findings of other studies, that found improved disordered eating after surgery (35, 66). Our findings suggest that bariatric surgery itself cannot be viewed as an intervention for disorder eating attitudes and behaviour, therefore psychological interventions designed to help adolescents in this matter could be useful. Some studies in adults found combination of lifestyle interventions and cognitive behaviour treatment as successful interventions for weight loss (67, 68).

We predicted that adolescents with fewer emotional and behavioural problems, better self-image and body satisfaction, and fewer disturber eating attitudes and behaviours will achieve greater weight loss. Our predictions were only partly confirmed by our results, that showed fewer overeating episodes and lower body satisfaction at baseline predicted successful weight loss 12 months after implantation of the device. Our findings are in accordance with previously published studies, that reported more frequent binge eating episodes as predictor of poorer weight loss after bariatric surgery (38, 46), but in contrast to others, where those association were not found (35, 40–43). We can assume that adolescents with fewer overeating episodes have more self-control than the ones with more overeating episodes, therefore they can control themselves better and are more adhere with dieting plans.

Unexpectedly, we observed that adolescents with higher body satisfaction lost less weight than others. Presented findings are in contrast with study where adolescents with better self-esteem have greater reduction of BMI 2 years post-surgery, while 5-years post-surgery relationship between self-esteem and weight loss was not significant any more (35). We suspect, that not overall self-esteem, but specifically but poorer satisfaction with body motivate adolescents to introduce some changes into their eating behaviours and other aspects of life, exercising, problem solving.

In addition, we observed that adolescents with more depressive symptoms, higher body satisfaction and more food restraining behaviour, showed lower probability of achieving weight loss if they had more overeating episodes. This finding suggests that some psychological variables, like depressive symptoms and food restraining, themselves does not have significantly strong effect on weight loss, but in combination overeating episodes does. Our results support the theory that mildly elevated symptoms of depression do not have effect on post-operative weight loss (44), while worse clinical manifestations (depression as psychiatric disorder) does (43). Similar, food restraining alone did not predict weight loss, but in combination with overeating become significant predictor. This raises the importance of evaluation of restraining feelings, especially as we observe higher levels of restraining at time of device removal in adolescent who did not respond for follow-up invitation year later.

Moreover, overeating episodes was one of the predictors with highest risk for unsuccessful weight loss and was moderating all significant interactions in simple slope analysis. Considering the fact, that overeating episodes did not improve after the procedure, which potentially shows how resistant these kind behaviours are, including evaluation and treatment of disorder eating in clinical setting is even of greater importance, specifically for adolescents with combined depressive symptomatology, higher food restraint, and higher body satisfaction.

Present findings should be considered within the context of several limitations. Firstly, groups were not randomised, which probably resulted in significant differences at baseline measures between control group and experimental group. Therefore, statistical analysis to correct baseline divergences was chosen. It is also not known whether the psychological characteristics of adolescents with severe obesity who decide for bariatric procedures are different in those seeking conservative treatment alternatives. Secondly, the small sample size might have prevented us from detecting important differences between the groups and reliable comparison on the basis of gender. Additionally, the attrition rate may have influenced our findings. Finally, psychological data were collected through self-report questionnaires, where there might be a risk of over- or under-reporting psychological distress.

We can conclude that specific psychological factors can predict how successfully adolescents will lose weight. Therefore, detailed and in-depth psychological evaluation, including estimation of disorder eating behaviours and attitudes, emotional and behavioural problems, and body satisfaction should be an essential part of pre-operative assessment, especially in adolescents. Presence of risk factors, like disturbed eating or depressive symptoms should not be necessary exclusion criteria for bariatric surgeries, rather the focus for additional psychological interventions (36). Some negative emotional states, like body dissatisfaction could be also a motivational factor, helping adolescents to healthier life. Integration of clinical psychologist or mental health professional, who recognised week points of individual adolescent, provide additional support, and promote post-operative adherence in the bariatric process could be crucial for success (24). In addition, psychological interventions specifically tailored to the adolescents, who are at risk for suboptimal weight loss in bariatric processes, should be included as part of the pre- and post-operative treatment plan to achieve successful weight loss.

Presented findings add to the knowledge in the research field of bariatric procedures in adolescents. Psychological outcomes in minimally invasive bariatric procedures are favourable and comparable to the outcomes of more invasive procedures in adolescents and adults. Results highlight the importance of psychological characteristic of the patients and multidisciplinary approach. Nonetheless, additional research with bigger sample size and longer follow-up is needed to evaluate the long-term effects of psychological aspect in minimally invasive bariatric procedures in adolescents.

In conclusion, following the reversible bariatric procedure, improvements of psychological (emotional and behavioural) factors were found in adolescents with severe obesity. Improvements were comparable to previously published results in non-reversible surgical bariatric procedures, adding a novel supplementary value to minimally invasive bariatric procedures. Reassuringly, most of the favourable psychological changes remained stable at follow-up, 12 months following device removal. Furthermore, psychological predictors of successful weight loss were identified, showing the greatest importance of eating behaviours and body satisfaction in successful weight loss. These findings highlight the importance of detailed psychological pre- and post-procedure assessment to identify potential difficulties, help achieve successful weight loss, and improve mental health status of adolescents with severe obesity.

## Data Availability Statement

The raw data supporting the conclusions of this article will be made available by the authors, without undue reservation.

## Ethics Statement

The studies involving human participants were reviewed and approved by the Republic of Slovenia National Medical Ethics Committee. Written informed consent to participate in this study was provided by the participants' legal guardian/next of kin.

## Author Contributions

SK, PK, and TB designed the study. SK, AB, NH, TB, MH, RO, and PK were involved in the acquisition and interpretation of data. The first draught of the paper was written by SK, with the support of NH and PK. SK is the guarantor of this work and, as such, had full access to all the data in the study and takes responsibility for the integrity of the data and the accuracy of the data analysis. All authors contributed to the conception of the work. All authors contributed to and approved the final version of the manuscript.

## Conflict of Interest

The authors declare that the research was conducted in the absence of any commercial or financial relationships that could be construed as a potential conflict of interest.
